# Influence of synbiotic supplementation on performance, fecal consistency, cecal microbiome, and egg quality of hens during late laying phase

**DOI:** 10.1016/j.psj.2026.106851

**Published:** 2026-03-22

**Authors:** Koonphol Pongmanee, Konkawat Rassmidatta, Ting-Yu Lee, Jin-Seng Lin, Chanporn Chaosap, Kazeem D. Adeyemi, Yuwares Ruangpanit

**Affiliations:** a Department of Animal Science, Faculty of Agriculture at Kamphaeng Saen, Kasetsart University, Kamphaeng Saen Campus, Nakhon Pathom 73140, Thailand; b Synbio Tech Inc., Kaohsiung 821011, Taiwan; c Department of Agricultural Education, School of Industrial Education and Technology, King Mongkut’s Institute of Technology Ladkrabang, Bangkok 10520, Thailand; d Department of Animal Production, Faculty of Agriculture, University of Ilorin, PMB 1515, Ilorin, Kwara State, Nigeria

**Keywords:** Alpha diversity, Cracked egg, Fecal ammonia, Lachnospiraceae, Shell thickness

## Abstract

Hens in the late laying phase often experience age-related physiological constraints that may reduce production efficiency, eggshell quality, and welfare. This study evaluated the effects of dietary synbiotic supplementation on laying performance, egg quality, fecal characteristics, and the cecal microbiome of late-phase laying hens. A total of 240 Lohmann Brown hens (50 weeks old) were distributed into 20 replicates and randomly assigned to a corn–soybean control diet or the same diet supplemented with 100 mg/kg synbiotic (SYNLAC Prime®) for 22 weeks. Synbiotic supplementation did not affect egg production or egg mass but significantly reduced feed intake (*P* = 0.036). It also improved eggshell quality by decreasing the proportion of cracked eggs (*P* = 0.014) and increasing eggshell weight (*P* = 0.049) and shell thickness (*P* = 0.031). Fecal score and moisture content were not affected; however, synbiotic-fed hens showed lower fecal ammonia concentration (*P* = 0.033). Synbiotic supplementation increased both alpha and beta microbial diversity in the cecum. While microbial composition at higher taxonomic levels was largely unchanged, the relative abundances of Ruminococcaceae and Lachnospiraceae increased, whereas those of Bacteroidaceae and Clostridiaceae decreased. At the genus level, synbiotic supplementation increased *Faecalibacterium, Ruminococcus*, and *Lactobacillus*, while reducing *Bacteroides* and *Alistipes*. Functional prediction analysis indicated that synbiotic supplementation upregulated 12 cecal metabolic pathways and downregulated two pathways. Overall, synbiotic supplementation improved eggshell quality, promoted beneficial gut microbial populations, and reduced fecal ammonia emissions, suggesting its potential as a dietary strategy to support productivity and gut health in hens during the late laying phase.

## Introduction

The global demand for poultry products, particularly eggs, continues to rise as they serve as an affordable and high-quality source of animal protein (Guyonnet, 2023). This growing demand exerts pressure on the laying hen industry to optimize production efficiency and maintain consistent egg quality throughout the laying cycle. However, sustaining productivity and egg quality beyond the peak laying period remains a major challenge. Hens in the late laying cycle commonly experience age-associated physiological and structural constraints that can alter production efficiency, egg integrity, and welfare (Liu et al., 2023; Wang et al., 2019).

Although egg size typically increases with age, the functional capacity of the shell gland does not expand proportionally, often resulting in a reduced shell-to-egg surface area coverage and a progressive decline in eggshell material accretion (Attia et al., 2020; Liu et al., 2023). This can weaken shell ultrastructure and shell membrane elasticity, leading to a higher incidence of cracked eggs and greater handling losses during collection and transport.

In parallel, aging layers undergo depletion of medullary bone and systemic calcium buffering reserves, contributing to osteoporosis, bone fragility, and elevated susceptibility to metabolic disorders in intensive systems (Attia et al., 2020; Hao et al., 2021; Liu et al., 2023). Reductions in digestive efficiency and greater protein fermentation in the gut with advancing age may increase fecal ammonia production compromising aerial environment and welfare (Lio et al., 2023). Furthermore, late-cycle hens exhibit immune senescence and microbiota restructuring risks, making them more vulnerable to inflammatory stress and dysbiosis (Chen et al., 2023; Gao et al., 2025; Wei et al., 2025; Xu et al., 2023).

Previously, antibiotic growth promoters are widely used to enhance productivity and maintain gut health in poultry (Insawake et al., 2025). However, concerns about antibiotic residues in eggs (Owusu-Doubreh et al., 2023), the emergence of antimicrobial resistance (Bava et al., 2024), and disruptions to the intestinal microbiota (Zhan et al., 2025) have led to strict regulations limiting their use. This shift has created an urgent need for safe and effective alternatives capable of supporting health and performance in laying hens without compromising food safety. Probiotics (Liu et al., 2023; Wang et al., 2021; Xu et al., 2023) and prebiotics (Adhikari et al., 2018; Elkomy et al., 2023; Youssef et al., 2024) have emerged as promising candidates, with numerous studies demonstrating improvements in laying performance, feed efficiency, and egg quality.

Synbiotics, combinations of probiotics and prebiotics, aim to achieve synergistic effects by enhancing the survival and activity of beneficial gut microbes. While several studies have reported improved gut microbiota balance, egg production, and egg quality with synbiotic supplementation (Obianwuna et al., 2023; Salem and El-Dayem, 2024; Sjofjan et al., 2020; Song et al., 2022), findings remain inconsistent, likely due to differences in synbiotic composition, microbial strains, dosage, and the age of birds. Moreover, limited information is available on the use of synbiotics during the late laying phase, a period marked by physiological decline. Modulating the intestinal microbiota may offer a strategy to counter late-phase challenges in hens (Dai et al., 2022; Wei et al., 2025). Therefore, we hypothesized that supplementation with a consortium of probiotics and maltodextrin would modulate the cecal microbiome and help mitigate physiological constraints in late-cycle hens. This study evaluated the effects of dietary synbiotics on performance, fecal characteristics, cecal microbiome, and egg quality in laying hens during the late production period.

## Materials and methods

### Ethics statement

All experimental procedures were reviewed and approved (Approval No. ACKU67-AGK-006) by the Institutional Animal Care and Use Committee of Kasetsart University.

### Hens, management, and experimental treatments

A total of 240 Lohmann Brown laying hens, aged 50 weeks, were utilized in this study. The hens were distributed into 24 replicates (12 hens per replicate), with three hens housed per cage (43 × 50 × 40 cm). The replicates were assigned at random to two dietary groups:

1. Control – a corn–soybean meal basal diet;
2. Synbiotic – the basal diet supplemented with 100 mg/kg synbiotic (SYNLAC Prime®, Synbio Tech Inc., Taiwan) containing *Pediococcus pentosaceus* (≥1.0 × 10^8^ cell/g), *Lactiplantibacillus plantarum* (≥5.5 × 10^9^ CFU/g), *Enterococcus faecium* (≥1.5 × 10^9^ CFU/g), *Lactobacillus acidophilus* (≥1.0 × 10^7^ CFU/g), and *Bacillus subtilis*) (≥1.0 × 10^9^ CFU/g), with maltodextrin as the carrier.

All hens were housed in an environmentally controlled layer house equipped with a mechanical ventilation system and evaporative cooling. Each cage was fitted with an automatic nipple drinker and a trough feeder, and allowed approximately equal space per hen in accordance with the Lohmann Brown management guide. The hens were subjected to a lighting schedule of 16 hours of light and 8 hours of darkness per day throughout the experimental period. Ambient temperature and relative humidity were recorded daily and maintained within the optimal range recommended for laying hens. Routine management practices were implemented according to farm standards. The trial lasted from 50 to 72 weeks of age, was divided into 11 two-week periods, and followed a single-phase feeding program.

All diets were formulated to meet the nutrient requirements of Lohmann Brown laying hens as recommended by the breeder manual. Feed was provided in mash form, and both feed and water were offered *ad libitum* throughout the study period. Representative samples of each diet were collected and analyzed for proximate composition, calcium, and phosphorus following the procedures of the AOAC (2019). Crude fiber was determined using the Weende method. The ingredient composition and nutrient profiles of the experimental diets are presented in Table 1, Table 2, respectively.

**Table 1:** Ingredient composition and calculated analysis of basal diet.

Ingredient	Control diet
Corn	57.71
Rice bran	1.51
Crude rice bran oil	0.65
SBM 46 % Crude protein)	27.10
L-lysine	0.18
DL-methionine	0.38
Choline chloride (60 %)	0.02
Monocalcium phosphate (21 % Phosphorus)	1.43
Calcium carbonate	10.39
Salt	0.37
Premix^1^	0.25
Non-starch polysaccharide degrading enzyme	0.01
Calculated analysis	
ME for poultry (kcal/kg)	2,679.31
Protein (%)	16.95
Ash (%)	2.00
Fat (%)	4.02
Fiber (%)	3.19
Calcium (%)	4.22
Total phosphorus (%)	0.66
Avail. phosphorus (%)	0.40
Salt (%)	0.44
Lysine (%)	1.05
Methionine (%)	0.65
Methionine + Cystine (%)	0.92
Choline (%)	1,797.03
Sodium (%)	0.16
Linoleic acid (%)	1.65

1 The laying hen premix (1 kg) contains vitamin A 4,000,000 IU, vitamin D3 1,000,000 IU, and vitamin E 9,000 IU, together with vitamin K3 (menadione) 1.2 g, vitamin B1 (thiamin) 0.4 g, vitamin B2 (riboflavin) 1.6 g, vitamin B6 (pyridoxine) 1.2 g, vitamin B12 0.01 g, niacin 12 g, pantothenic acid 4 g, folic acid 0.2 g, and biotin 0.04 g. The premix also provides essential trace minerals, including manganese 40 g, zinc 24 g, iron 10 g, iodine 0.2 g, copper 2 g, selenium 0.08 g, and cobalt 0.02 g, along with 10.3 g of feed additives and 2 g of preservatives, with an inert carrier added to complete 1 kg.

**Table 2:** Analyzed nutrient composition of experimental diets.

	Control	Synbiotic
Dry mater (%)	88.95	88.72
Protein (%)	16.35	16.58
Fat (%)	2.90	2.90
Fiber (%)	3.15	3.17
Ash (%)	12.94	12.92
Calcium (%)	4.04	4.10
Phosphorus (%)	0.61	0.60
GE (kcal/kg)	3,481.68	3,475.53

### Laying hen performance

Body weight of all hens was recorded at the beginning and end of the trial. During each laying period, the number of eggs produced, including cracked and dirty eggs, and individual egg weights were recorded. These data were used to calculate average egg weight, hen-house egg production (HH, %), hen-day egg production (HD, %), egg mass (g), and the proportions of cracked and dirty eggs. Feed offered and refusals were recorded periodically for each replicate to determine feed intake per hen and calculate feed conversion ratio (FCR). Mortality was monitored by recording the number of dead hens, and the number of culled hens (those showing poor health, sickness, underweight, or leg problems) was used to determine the culling rate. Ambient temperature and relative humidity were measured daily.

### Egg quality

Egg quality was assessed using four eggs per replicate at each laying period. Eggs were evaluated using a digital egg tester (NABEL DET6500, Kyoto, Japan) to determine egg weight, albumen height, Haugh unit, yolk height, yolk diameter, yolk index, yolk colour, and eggshell breaking strength. Yolk weight was calculated by subtracting the shell and albumen weights from the total egg weight. For shell measurements, eggshells were dried overnight at 30 ± 2°C before weighing. The dried shells were then used to determine eggshell thickness. After removal of the shell membrane, shell thickness was measured at three locations (sharp end, equator, and blunt end) using a digital micrometre (Mitutoyo, Japan). The mean of the three measurements was calculated as the eggshell thickness for each egg.

### Fecal analysis

At 72 weeks of age, fresh excreta samples were collected from each cage for the determination of fecal consistency score, excreta moisture content, and ammonia concentration. Fifty grams of excreta were collected from all replicate cages between 9.00 am to 12.00 pm, pooled by cage, and a representative subsample was taken for further analysis. The moisture content of the excreta was determined by AOAC (2019) method. Ammonia concentration in the collected excreta was quantified according to Mi et al. (2019) procedure. Fecal consistency was scored using a four‐point numerical scale adapted from Zhu et al. (2020) as follows:

*Score 1 = hard, dry, and pellet-like feces*
*Score 2 = firm, well-formed feces*
*Score 3 = soft, moist feces that still retain shape*
*Score 4 = soft, unformed feces*

### Cecal microbiome analysis

At week 72, one hen per replicate was randomly selected and euthanized, and cecal contents were collected and stored at −80°C for 16S rRNA sequencing. Genomic DNA was extracted using the ZymoBIOMICS™ DNA Miniprep Kit (Zymo Research, USA), and samples were preserved in DNA/RNA Shield™ to prevent nucleic acid degradation. DNA quality and concentration were assessed using a NanoDrop™ 2000 Spectrophotometer, QFX Fluorometer (DeNovix), and Bioanalyzer 2100 system (Agilent Technologies).

The V3–V4 region of the 16S rRNA gene was amplified using specific forward (5′-TCGTCGGCAGCGTCAGATGTGTATAAGAGACAGCCTACGGGAGGCAGCAG-3′) and reverse primers (5′-GTCTCGTGGGCTCGGAGATGTGTATAAGAGACAGATTACCGCGGCTGCTGG-3′). PCR reactions (25 µL) contained Phusion Hot Start II HF Master Mix, 0.2 µM primers, and ∼10 ng template DNA. Thermocycling conditions consisted of 95°C for 3 min; 25 cycles of 95°C for 30 s, 55°C for 30 s, and 72°C for 30 s; followed by a final extension at 72°C for 5 min. Amplicons (∼550 bp) were verified on 1.5 % agarose gels, purified using AMPure XP beads, and indexed with the Nextera XT Index Kit. Libraries were prepared with the NEBNext® Ultra™ II DNA Library Prep Kit, pooled, quantified, diluted to 2 nM, spiked with 2 % PhiX control, and adjusted to a final loading concentration of 1.5 pM.

Sequencing was performed on an Illumina MiSeq platform (2 × 151 bp paired-end). Raw reads were processed in QIIME2 (version 2020.11) using the DADA2 pipeline for quality filtering, denoising, and chimera removal. Taxonomic assignment was conducted against the PKSSU 4.0 database (Yoon et al., 2017). Operational taxonomic units (OTUs) were identified using open-reference OTU picking at 99 % similarity. Alpha diversity indices (Chao1, phylogenetic diversity, Shannon) and beta diversity (weighted UniFrac) were calculated, with beta-diversity visualized through principal coordinates analysis (PCoA) based on 42,106 rarefied reads per sample.

The metabolic potential of the cecal microbiota was predicted using PICRUSt2 via the q2-picrust2 plugin in QIIME2 (version 2019.10). Predicted gene functions were annotated against the KEGG database, and KEGG orthologs were grouped and normalized by sample. MetaCyc was used to reconstruct metabolic pathways. Functional outputs and pathway-level differences (including amino acid metabolism) were analyzed using R (vegan, ggplot2) and STAMP (v.2.1.3). Figures were generated with GraphPad Prism (version 10.4.0).

### Statistical analysis

Performance, egg quality, and excreta parameter data were analyzed using the t-test procedure. The replicate cage served as the experimental unit for performance, feed intake, and excreta measures, while egg-quality data were averaged per cage before analysis. For the ordinal fecal consistency score (four-point scale), a non-parametric method appropriate for ordinal data was applied: score frequencies were summarized per cage, and treatment differences were evaluated using the Kruskal–Wallis test. When significant effects were detected, mean treatment scores were presented, and pairwise comparisons adjusted for ties were conducted.

Alpha diversity was compared between groups using a non-parametric t-test with 999 permutations. Beta diversity was assessed by principal coordinates analysis (PCoA) based on weighted UniFrac distances, followed by permutational multivariate analysis of variance using the Adonis function in R (version 4.2.2) with the *vegan* package (version 2.5-7). Differentially abundant OTUs were identified using negative binomial modeling in DESeq2. Pairwise comparisons of microbial metabolic pathways were conducted using a two-sided Welch’s t-test.

## Results

### Laying performance

Hen-house and hen-day egg production, average egg weight, egg mass, FCR, dirty egg percentage, and livability were not significantly affected by synbiotic supplementation (Table 3). However, the proportion of cracked eggs was markedly reduced in the synbiotic group compared to the control (*P* = 0.014). In addition, hens receiving the synbiotic diet consumed less feed than the control hens (*P* = 0.036).

**Table 3:** Performance of laying hens supplemented with a synbiotic during 50 to 72 weeks of age.

	Control	Synbiotic	SEM	*P*-value
Hen-house egg production (%)	94.67	94.33	0.72	0.405
Hen-day egg production (%)	94.84	94.41	0.73	0.383
Average egg weight (g)	63.31	63.68	0.24	0.292
Egg mass (g)	60.00	60.08	0.48	0.469
Cracked egg (%)	0.065	0.005	0.01	0.014
Dirty egg (%)	0.175	0.005	0.09	0.172
Feed intake (g)	120.61	118.67	0.48	0.036
Feed conversion ratio	2.013	1.981	0.01	0.226
Livability (%)	99.99	100	0.002	0.172

SEM, standard error of mean.

### Egg quality

No significant differences were observed in average egg weight, eggshell breaking strength, albumen height, Haugh unit, yolk color, yolk weight percentage, albumen weight or percentage (Table 4). However, synbiotic supplementation resulted in a significant increase in eggshell weight (*P* = 0.049) and shell thickness (*P* = 0.031) compared to the control group.

**Table 4:** Egg quality of laying hens supplemented with a synbiotic during 50 to 72 weeks of age.

	Control	Synbiotic	SEM	*P*-value
Average egg weight (g)	62.53	62.73	0.13	0.232
Eggshell breaking strength (N)	50.87	51.42	0.44	0.269
Albumen height (mm)	7.56	7.70	0.05	0.077
Haugh unit	85.36	86.40	0.32	0.078
Yolk color	8.38	8.33	0.03	0.144
Eggshell weight percentage (%)^1^	13.72	14.04	0.08	0.049
Yolk weight percentage (%)^2^	26.72	26.66	0.11	0.401
Albumen weight percentage (%)^3^	59.50	59.31	0.16	0.308
Shell thickness (mm)	0.367	0.371	0.001	0.031

SEM, standard error of mean.

1 $Eggshell\;weight\;\left(\%\right)=\left(\frac{Eggshell\;weight}{Whole\;egg\;weight}\right)\;x\;100.$

2 $Yolk\;weight\;\left(\%\right)=\left(\frac{Yolk\;weight}{Whole\;egg\;weight}\right)\;x\;100.$

3 $Albumen\;weight\;\left(\%\right)=\left(\frac{Albumen\;weight}{Whole\;egg\;weight}\right)\;x\;100.$

### Fecal analysis

No significant differences were observed in fecal score (*P* = 0.059) or fecal moisture content (*P* = 0.459) between the control and synbiotic groups (Table 5). In contrast, synbiotic supplementation reduced fecal ammonia concentration compared to the control group (*P* = 0.033).

**Table 5:** Fecal properties of laying hens supplemented with a synbiotic at 72 weeks of age.

	Control	Synbiotic	SEM	*P*-value
Fecal score (%)	2.17	1.93	0.09	0.059
Fecal moisture (%)	74.91	74.82	0.56	0.459
Fecal ammonia (ppm)	1,631.87*	1,500.41	51.96	0.033

SEM, standard error of mean.

### Ceca microbiome

**Fig. 1:**
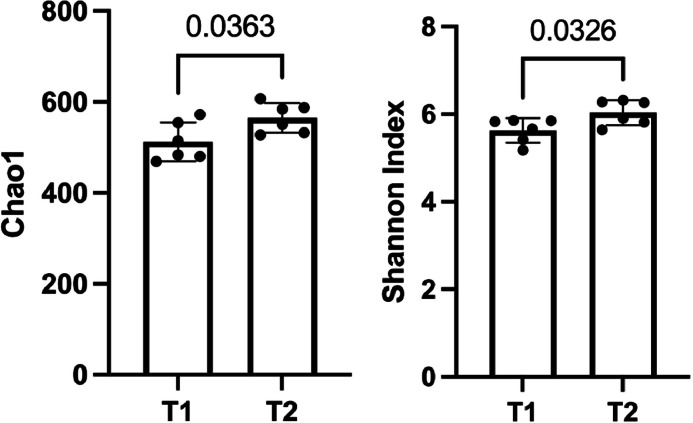
Alpha diversity (Chao1 and Shannon Index) of caeca microbiota of laying hens supplemented with a synbiotic. TI, corn-soybean basal diet (control), T2, basal diet supplemented with a synbiotic.

The Chao1 and Shannon indices revealed significant differences in microbial richness and diversity between treatments (Fig. 1). Synbiotic-fed hens exhibited higher Chao1 (*P* = 0.036) and Shannon diversity index (*P* = 0.033) compared with the control hens.

**Fig. 2:**
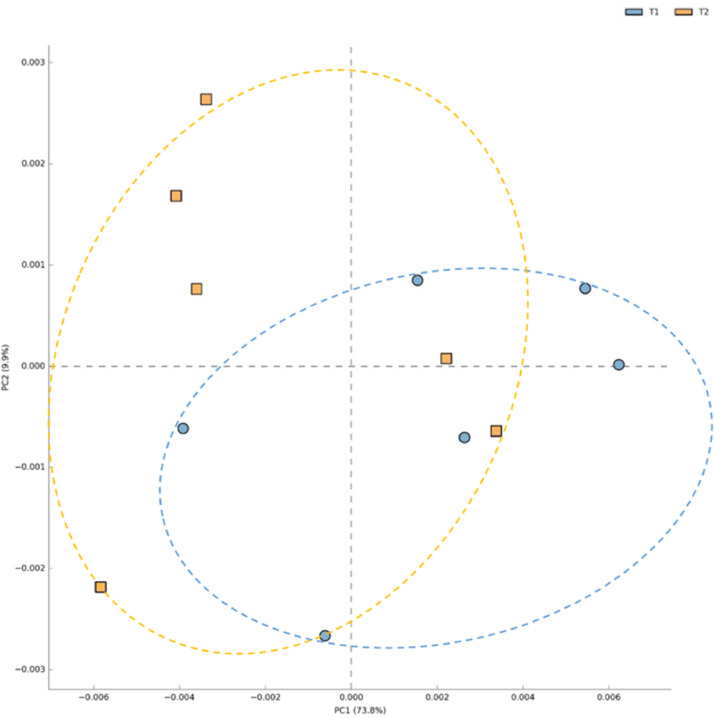
Beta diversity of caeca microbiota of laying hens supplemented with a synbiotic. TI, corn–soybean basal diet (control), T2, basal diet supplemented with a synbiotic.

The Principal Coordinate Analysis (PCoA) based on beta diversity metrics revealed a low but distinct clustering pattern between the control and synbiotic groups, a clear difference in the overall microbial community composition (β-diversity) of the cecal microbiota (Fig. 2). The spatial separation between the two treatment groups showed a significantly altered microbial structure in the laying hens.

**Fig. 3:**
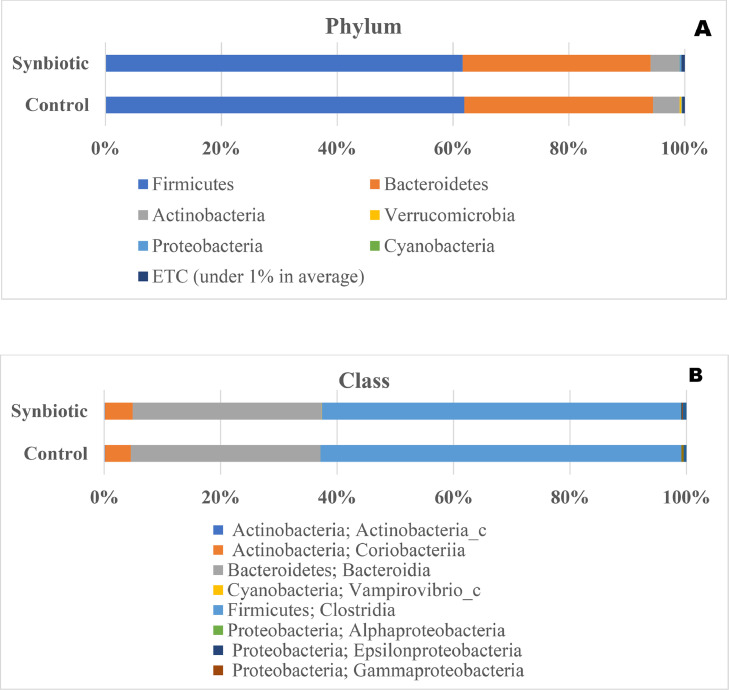

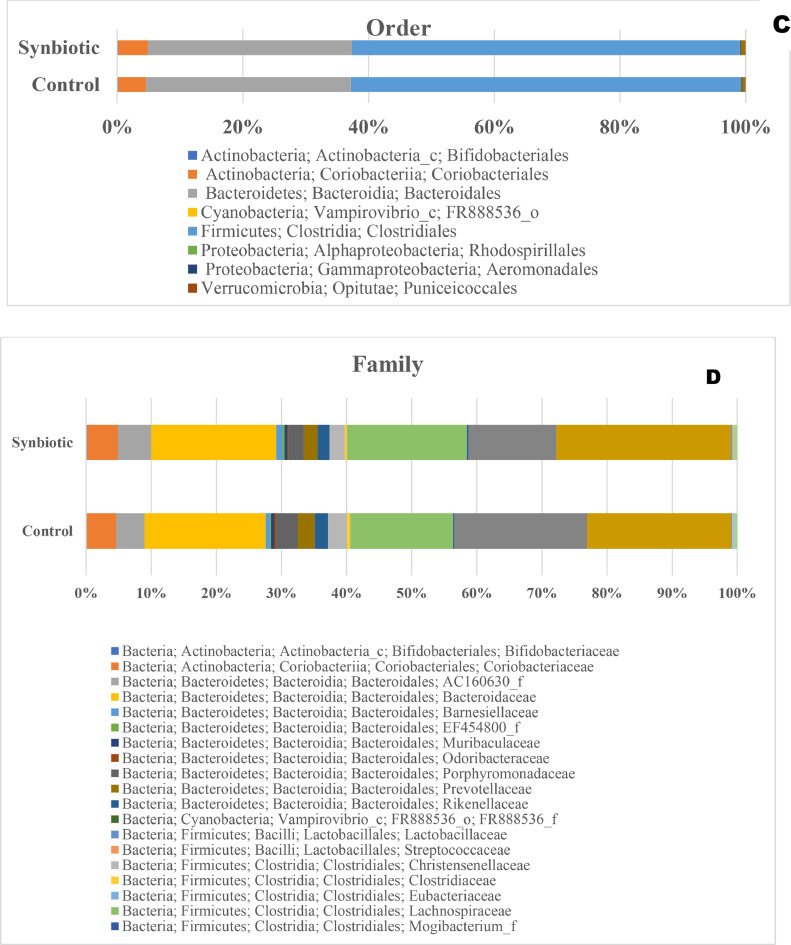

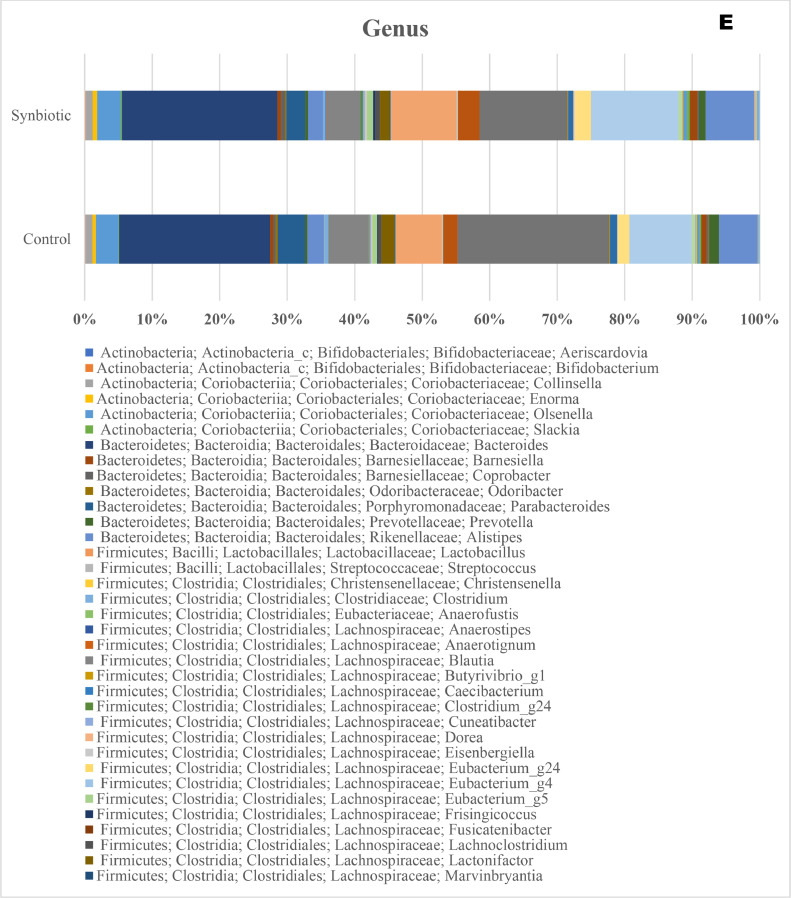

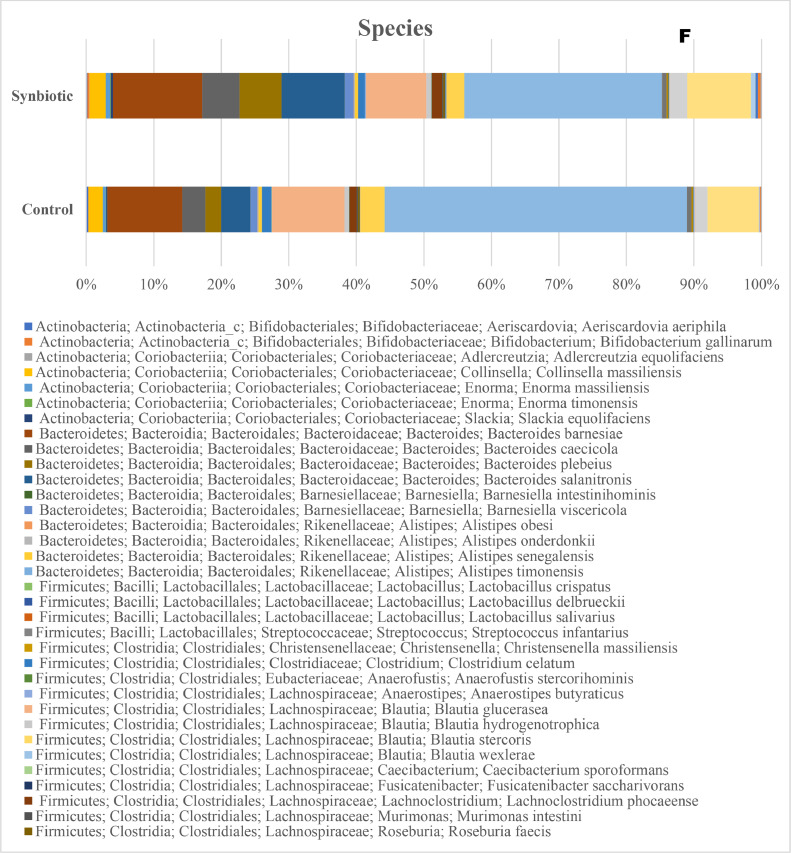
Taxa distribution at the phylum (A), class (B), order (C), family (D), genus (E), and species (F) of caeca microbiota of hens supplemented with a synbiotic.

The taxonomic distributions of the cecal microbiota at the phylum, class, order, family, genus, and species levels of laying hens are presented in Fig. 3. At the phylum level (Fig. 3A), the dominant bacterial taxa in both treatments were Firmicutes and Bacteroidetes, followed by Actinobacteria, Verucomicrobia, Proteobacteria, and Cyanobacteria and their relative abundances did not differ between diets. At the class level (Fig. 3B), Clostridia and Bacteroidia were the predominant classes across treatments, and their relative abundance was not influenced by diets. At the order level (Fig. 3C), the major taxa were Clostridiales, Bacteroidales, and Verrucomicrobiales, and their relative abundances were not affected by dietary synbiotics.

At the family level (Fig. 3D), the cecal microbiota was dominated by Ruminococcaceae, Lachnospiraceae, and Bacteroidaceae. The synbiotic hens exhibited higher relative abundance of Ruminococcaceae and Lachnospiraceae, and lower abundance of Bacteroidaceae, Clostridiaceae and Peptostreptococcaceae. At the genus level (Fig. 3E), synbiotic supplementation increased the relative abundance of *Faecalibacterium, Ruminococcus*, and *Lactobacillus*, while the proportions of *Bacteroides* and *Alistipes* were reduced. At the species level (Fig. 3F) the dominant species were affiliated with the Firmicutes and Bacteroidetes phyla, but their relative proportions varied. The synbiotic hens exhibited higher abundance of *Faecalibacterium prausnitzii, Ruminococcus bromii, Lactobacillus salivarius,* and *Butyricicoccus pullicaecorum* and a lower abundance of *Bacteroides fragilis, Alistipes onderdonkii*, and *Parabacteroides distasonis*.

**Fig. 4:**
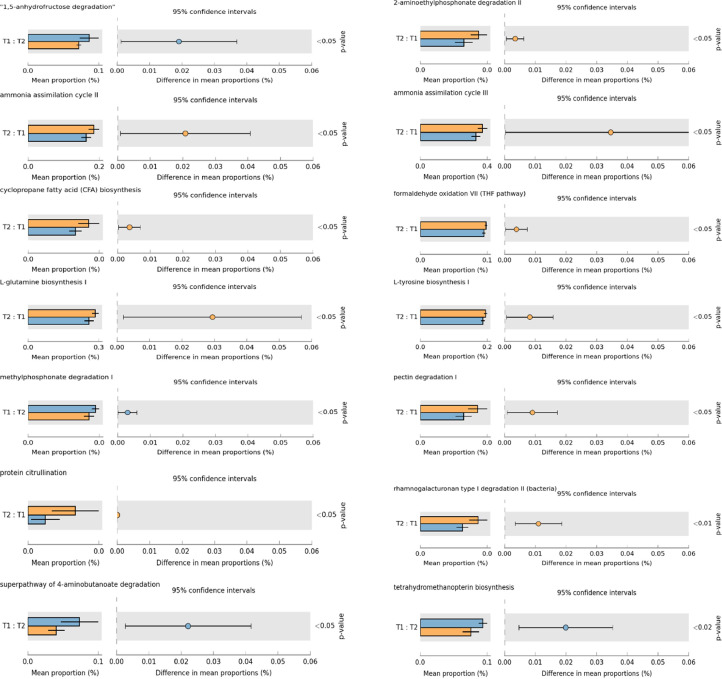
Metabolic pathways in caeca of laying hens supplemented with synbiotics. T1, Corn-soybean basal diet T2, Basal diet supplemented with a synbiotic.

Synbiotic supplementation modulated several microbial metabolic pathways in layer cecum (Fig. 4). Synbiotic supplementation upregulated 2-aminoethylphosphate degradation II (*P* = 0.007), ammonia assimilation cycle II (*P* = 0.020), ammonia assimilation cycle III (*P* = 0.035), cyclopropane fatty acid, biosynthesis (*P* = 0.005), formaldehyde oxidation VII (*P* = 0.005), L-glutamine biosynthesis I (*P* = 0.030), L-tyrosine biosynthesis I (*P* = 0.010), pectin degradation 1 (*P* = 0.010), protein citrullination (*P* = 0.001), and rhamnogalacturonan type 1 degradation II (Bacteria) (*P* = 0.010) pathways and downregulated 1, 5-anhydrofructose degradation (*P* = 0.020), methylphosphate degradation 1 (*P* = 0.005), 4-aminobutanoate degradation (*P* = 0.022), and tetrahydromethanopterin biosynthesis (*P* = 0.020) pathways.

## Discussion

Synbiotic supplementation did not influence the fundamental determinants of laying performance, as no significant differences were detected for production rate, egg weight, egg mass, FCR, dirty egg incidence, or livability. This aligns with reports that synbiotics primarily modulate gastrointestinal functionality rather than directly altering endocrinological drivers of ovulation or oviposition. For instance, Song et al. (2022) observed no improvement in laying performance when *Lactobacillus plantarum, Bacillus subtilis*, and fructo-oligosaccharides were used. Conversely, some studies found improvements in laying rate, FCR, and egg size with synbiotic inclusion (Obianwuna et al., 2023; Tang et al., 2017). Such variability in results may be due to differences in synbiotic composition, dosage, strain viability, and the production phase of the birds.

Despite the performance-neutral response of production indices, synbiotics conferred a significant reduction in cracked egg proportion. The mechanism is plausibly linked to improved mineral bioavailability and microbiota-mediated modulation of gut metabolites that influence eggshell structural matrix and shell membrane stability. Enhanced calcium absorption associated with synbiotic-supported microbiota, as previously demonstrated in hens fed synbiotic blend (Abdelqader et al., 2013; Salem and El-Dayem, 2024), may increase shell fracture resistance without necessarily changing egg size or total mass. This is of commercial relevance because cracked eggs carry a higher risk of microbial ingress and economic downgrading. Consistently, the addition of synbiotic composed of 0.02 % *Clostridium butyricum* and 0.6 % fructooligosaccharides lowered the number of damaged eggs in layers (Obianwuna et al., 2023).

Synbiotic-fed hens consumed less feed while maintaining comparable egg mass and feed conversion ratio, suggesting improved digestive efficiency and nutrient utilization. Synbiotics can enhance gut microbial balance, stimulate digestive enzyme secretion, and improve intestinal morphology, thereby increasing nutrient utilization and microbial fermentation efficiency, which allows birds to meet their nutrient requirements at lower feed intake levels (Zhou et al., 2023). Although not strong enough to shift FCR statistically, the feed reduction itself may translate to reduced cost per unit production if sustained over a production cycle. Comparable reductions in feed intake without compromised productive output were noted in layer trial integrating synbiotics (Sjofjan et al., 2020).

The evaluated internal egg quality traits, including albumen height, Haugh unit, yolk color, yolk proportion, and albumen proportion, remained statistically unchanged following synbiotic supplementation. This suggests that the synbiotic formulation did not modulate parameters largely governed by systemic nutrient deposition, such as albumen protein quality or pigment metabolism, likely because the basal diets already met the nutritional requirements of the layers. In agreement with these results, previous studies have also reported no effects of synbiotics on internal egg quality in chickens (Tang et al., 2015) and Pengging ducks (Kismiati et al., 2022).

Synbiotic supplementation improved eggshell weight and thickness, likely by enhancing intestinal integrity, reducing gut pH via short chain fatty acid (SCFA) production, and increasing calcium bioavailability for eggshell mineralization (Ahmed et al., 2023; Śliżewska et al., 2020). These changes strengthen the gut shell axis, facilitating increased deposition of CaCO_3_ in the uterus and resulting in heavier and thicker shells. The improved shell traits also support the earlier reduction in cracked eggs. Our findings cohere with those of Abdelqader et al. (2013), who reported that a synbiotic composed of *Bacillus subtilis* and inulin enhanced eggshell quality in layers. Similarly, Abdel-Wareth (2016) showed that supplementation of synbiotics, thymol, or their combination improved eggshell thickness and shell percentage in laying hens.

A significant environmental outcome of this study was the reduction in fecal ammonia concentration among the synbiotic-fed hens. Lower ammonia emissions are beneficial both for bird welfare and environmental sustainability. The reduction in ammonia likely reflects enhanced microbial fermentation and protein digestibility, as efficient microbial metabolism reduces undigested protein available for ammonia formation in the hindgut (Mi et al., 2019; Pan and Yu, 2014). Fecal moisture and consistency were unaffected, indicating stable intestinal function.

Synbiotic supplementation significantly enhanced cecal microbial diversity, as indicated by higher Chao1 richness and Shannon index values compared with the control. Increased α-diversity typically reflects a more heterogeneous and resilient intestinal ecosystem with greater functional redundancy and reduced susceptibility to dysbiosis (Insawake et al., 2024; Wang et al., 2020). This aligns with the expected effects of synbiotics, which promote beneficial microbial colonization and support microbial coexistence through complementary prebiotic–probiotic interactions (Prentza et al., 2022).

β-diversity analysis (PCoA) showed a modest but clear separation between the control and synbiotic groups, indicating that the synbiotic blend altered overall microbial structure rather than only a few taxa. The mild yet distinct clustering suggests that synbiotics reorganized community composition and relative abundance without causing major ecological disruption—a pattern typical in well-managed flocks where microbial additives enhance community complexity without causing drastic shifts (Rassmidatta et al., 2024).

The cecal microbiota remained compositionally typical of adult layers (Dai et al., 2022; Wang et al., 2020). At the phylum level, Firmicutes and Bacteroidetes predominated in both groups, indicating that synbiotic supplementation did not disrupt the established core microbiota. Similarly, dominant classes (Clostridia, and Bacteroidia) and orders (Clostridiales and Bacteroidales) were unaffected, suggesting that key fiber-fermenting functional groups remained stable. At the family level, the microbiota was largely composed of Ruminococcaceae, Lachnospiraceae and Bacteroidaceae. Synbiotic supplementation increased Ruminococcaceae and Lachnospiraceae while reducing Bacteroidaceae, Clostridiaceae and Peptostreptococcaceae. The enrichment of Ruminococcaceae and Lachnospiraceae is notable because these families are major butyrate producers that support epithelial integrity, antimicrobial peptide expression, immune regulation and calcium solubilization (Wang et al., 2020; Xiao et al., 2021). Enhanced butyrate-producing consortia may help explain the improved eggshell mass and reduced cracked eggs observed in this study, as SCFAs and lower gut pH promote efficient calcium transport to the shell gland (Dai et al., 2022; Jin et al., 2024; Śliżewska et al., 2020). The reductions in Bacteroidaceae, Clostridiaceae and Peptostreptococcaceae likely reflect competitive exclusion driven by the administered probiotic species (*Lactobacillus acidophilus, L. plantarum, Pediococcus pentosaceus, Enterococcus faecium* and *Bacillus subtilis*). These strains compete for nutrients, adhesion sites and fermentation substrates, and prebiotic components such as maltodextrin selectively promote SCFA-producing taxa, lowering luminal pH and creating conditions unfavorable for these families (Khan et al., 2020). Peptostreptococcaceae members include urease-active species involved in ammoniagenesis (Cao et al., 2003); their decline corresponds with the lower fecal ammonia concentration observed in the synbiotic-fed hens.

At the genus level, the synbiotic diet increased *Faecalibacterium, Ruminococcus* and *Lactobacillus*, while decreasing *Bacteroides* and *Alistipes*. These shifts reflect a microbial environment favoring fiber-degrading and lactic acid–producing taxa. *Lactobacillus* contributes to competitive exclusion and acidification, whereas *Faecalibacterium* and *Ruminococcus* generate butyrate, supporting epithelial integrity and reducing inflammation (Khan et al., 2020). Conversely, *Bacteroides* and *Alistipes*, often associated with protein fermentation and opportunistic behavior, declined likely due to increased SCFA production and competition for substrates (Dai et al., 2022; Sun et al., 2021). At the species level, synbiotic-fed hens showed higher abundances of *Faecalibacterium prausnitzii, Ruminococcus bromii, Lactobacillus salivarius* and *Butyricicoccus pullicaecorum*, and lower levels of *Bacteroides fragilis, Alistipes onderdonkii* and *Parabacteroides distasonis*. These increases reflect enhanced butyrate production and improved mucosal health. The reduced abundance of *Bacteroides* and *Alistipes,* taxa associated with inflammation and low-fiber conditions, suggests a shift toward a more balanced and health-promoting microbiota (Dai et al., 2022; Sun et al., 2021).

Synbiotic supplementation altered microbial metabolic potential. Upregulated pathways, including 2-aminoethylphosphate degradation II, pectin and rhamnogalacturonan degradation, and cyclopropane fatty acid biosynthesis, indicate enhanced utilization of complex carbohydrates, phospholipid derivatives and plant polysaccharides, consistent with increased *Faecalibacterium, Ruminococcus* and *Butyricicoccus*. Enhanced pectin and rhamnogalacturonan degradation suggest improved SCFA (especially butyrate) production. The upregulated ammonia assimilation pathways (cycles II and III) and increased L-glutamine and L-tyrosine biosynthesis indicate more efficient microbial nitrogen capture. This shift toward incorporating ammonia into amino acids provides a mechanistic basis for the reduction in fecal ammonia in synbiotic-fed hens. Reduced ammonia release also limits harmful nitrogenous metabolites associated with dysbiosis. Increased formaldehyde oxidation VII and protein citrullination further support detoxification and improved host–microbe interactions. In contrast, downregulation of 1,5-anhydrofructose degradation, 4-aminobutanoate degradation and tetrahydromethanopterin biosynthesis suggests reduced microbial stress responses, decreased amino acid breakdown and lower methanogenic potential. The suppression of tetrahydromethanopterin biosynthesis, a key cofactor in methane metabolism, indicates reduced activity of methane-associated bacteria often favored under protein-rich or dysbiotic conditions. The methylphosphate degradation I pathway is responsible for breaking down organophosphate compounds particularly methylphosphonate into utilizable phosphorus and carbon sources. The downregulation of this pathway in the synbiotic-fed hens indicates that the cecal microbiota became less reliant on phosphonate breakdown and shifted toward taxa that preferentially use carbohydrate-based energy sources. This interpretation aligns with the observed decrease in Bacteroides, Alistipes, and related taxa that possess phosphonate-metabolism genes, which likely contributed to the reduced expression of methylphosphate degradation pathways in the synbiotic group.

## Conclusions

Synbiotic supplementation during the late laying phase did not alter overall egg production but improved key aspects of egg quality by reducing cracked eggs and enhancing eggshell weight and thickness, while also lowering feed intake. Although fecal consistency remained unchanged, synbiotic-fed hens exhibited reduced fecal ammonia, indicating improved gut fermentation efficiency. The synbiotic also promoted a more diverse and beneficial cecal microbiome, characterized by increases in butyrate-producing taxa such as Ruminococcaceae, Lachnospiraceae, *Faecalibacterium*, and *Lactobacillus*, alongside reductions in potentially unfavorable bacterial families and species. Corresponding shifts in metabolic pathways further support functional enhancement of the cecal ecosystem. Collectively, these findings highlight the potential of synbiotics as a nutritional strategy to support gut health and enhance nitrogen utilization and eggshell quality in hens during the late laying period.

## CRediT authorship contribution statement

**Koonphol Pongmanee:** Resources, Methodology, Investigation. **Konkawat Rassmidatta:** Resources, Project administration, Methodology, Investigation, Formal analysis. **Ting-Yu Lee:** Resources, Methodology, Investigation. **Jin-Seng Lin:** Resources, Methodology, Investigation. **Chanporn Chaosap:** Resources, Methodology, Investigation. **Kazeem D. Adeyemi:** Writing – review & editing, Writing – original draft, Methodology, Formal analysis. **Yuwares Ruangpanit:** Supervision, Resources, Project administration, Methodology, Investigation, Funding acquisition, Conceptualization.

## Disclosures

The authors declare that they have no known competing financial interests or personal relationships that could have appeared to influence the work reported in this paper.
